# Validation of automated Alberta Stroke Program Early CT Score (ASPECTS) software for detection of early ischemic changes on non-contrast brain CT scans

**DOI:** 10.1007/s00234-020-02533-6

**Published:** 2020-08-28

**Authors:** Lennard Wolff, Olvert A. Berkhemer, Adriaan C. G. M. van Es, Wim H. van Zwam, Diederik W. J. Dippel, Charles B. L. M. Majoie, Theo van Walsum, Aad van der Lugt, for the MR CLEAN Investigators

**Affiliations:** 1 Department of Radiology & Nuclear Medicine, Erasmus MC, P. van Andel & L. Wolff, room Ne-515, Postbus 2040, 3000 CA Rotterdam, the Netherlands; 2 Department of Radiology & Nuclear Medicine, Amsterdam University Medical Centers, location AMC, Amsterdam, the Netherlands; 3 Department of Neurology, Erasmus MC, Rotterdam, the Netherlands; 4 Department of Radiology, Maastricht UMC+, Maastricht, the Netherlands; 5 Biomedical Imaging Group Rotterdam, Erasmus MC, Rotterdam, the Netherlands

**Keywords:** Stroke, Tomography, X-Ray Computed, Brain ischemia, Image Processing, Computer-Assisted, Software validation

## Abstract

**Purpose:**

In ASPECTS, 10 brain regions are scored visually for presence of acute ischemic stroke damage. We evaluated automated ASPECTS in comparison to expert readers.

**Methods:**

Consecutive, baseline non-contrast CT-scans (5-mm slice thickness) from the prospective MR CLEAN trial (*n* = 459, MR CLEAN Netherlands Trial Registry number: NTR1804) were evaluated. A two-observer consensus for ASPECTS regions (normal/abnormal) was used as reference standard for training and testing (0.2/0.8 division). Two other observers provided individual ASPECTS-region scores. The Automated ASPECTS software was applied. A region score specificity of ≥ 90% was used to determine the software threshold for detection of an affected region based on relative density difference between affected and contralateral region. Sensitivity, specificity, and receiver-operating characteristic curves were calculated. Additionally, we assessed intraclass correlation coefficients (ICCs) for automated ASPECTS and observers in comparison to the reference standard in the test set.

**Results:**

In the training set (*n* = 104), with software thresholds for a specificity of ≥ 90%, we found a sensitivity of 33–49% and an area under the curve (AUC) of 0.741–0.785 for detection of an affected ASPECTS region. In the test set (*n* = 355), the results for the found software thresholds were 89–89% (specificity), 41–57% (sensitivity), and 0.750–0.795 (AUC). Comparison of automated ASPECTS with the reference standard resulted in an ICC of 0.526. Comparison of observers with the reference standard resulted in an ICC of 0.383–0.464.

**Conclusion:**

The performance of automated ASPECTS is comparable to expert readers and could support readers in the detection of early ischemic changes.

**Electronic supplementary material:**

The online version of this article (10.1007/s00234-020-02533-6) contains supplementary material, which is available to authorized users.

## Introduction

In the treatment of acute ischemic stroke, the severity and extent of an ischemic stroke lesion could be used as one of the parameters to select eligible patients for endovascular treatment [1].[1] NCCT of the brain is the most widely used modality for assessment of early focal signs of ischemic damage in stroke patients. To quantify the extent of ischemia on NCCT, the Alberta Stroke Program Early Computed Tomography Score (ASPECTS) has been introduced. In ASPECTS, 10 brain regions are dichotomously scored on the presence of early ischemic stroke signs, resulting in a range of 0 to 10, with 1 point subtracted for any evidence of early ischemic change in each defined region on the CT scan [2]. ASPECTS scoring requires a high level of expertise to detect subtle changes on NCCT in the early phase of brain ischemia [3]. This expertise is not available in every center where stroke patients are presented. Consequently, there is considerable interrater variability [4–7]. Automated tools have been developed to counter these challenges [3, 8–11].

Siemens has developed a fully automated post-processing tool to score ASPECTS on NCCT [12, 13]. The performance of automated software in comparison to physicians should be tested before this software is used in clinical practice as aid for physicians. In this study, we evaluated Frontier ASPECTS software for the detection of early ischemic brain changes on NCCT scans acquired on a broad range of different CT scanners.

## Materials and methods

### Study design

We used image data from the Multicenter Randomized Clinical Trial of Endovascular Treatment for Acute Ischemic Stroke in The Netherlands (MR CLEAN, MR CLEAN Netherlands Trial Registry number NTR1804. Current Controlled Trials number, ISRCTN10888758), a prospective, consecutive study which was performed in 16 stroke centers in the Netherlands [14]. Detailed study methods and eligibility criteria were published previously [15].

Patients with an occlusion of the intracranial carotid artery, the M1/M2 segment of the middle cerebral artery or the A1/A2 segment of the anterior cerebral artery were included in the MR CLEAN trial (*n* = 500). ASPECTS or the severity and the extent of early ischemic changes were not used as exclusion criteria.

The MR CLEAN study protocol was approved by the central medical ethics committee of the Erasmus MC and the research board of each participating center. All patients or their legal representatives provided written informed consent before randomization.

### Imaging data and evaluation

As MR CLEAN was a multicenter trial, various CT scanner models had been used to obtain the NCCT of the brain which resulted in a heterogeneous dataset with scans from all major CT scanner manufacturers (GE Healthcare, Chicago, USA; Philips Healthcare, Amsterdam, The Netherlands; Siemens Healthineers, Erlangen, Germany; Toshiba, Tokyo, Japan). All patients with NCCT images with 5-mm slice thickness were included in the current study.

All baseline NCCT scans were evaluated for ASPECTS four times by expert readers who were unaware of the treatment group assignments and final outcome. The expert readers were blinded for all clinical information, except the clinically affected cerebral hemisphere.

To define a reference standard for ASPECTS, every CT scan was first rated by two expert readers from a pool of eight readers to produce a consensus score for every ASPECTS region (n = 10). In case of disagreement, a consensus score was provided by a third reader [16].

In addition, every CT-scan was rated by two expert readers from a second pool of nine readers to produce two individual ASPECTS, hereafter named as ASPECTS of observer 1 and observer 2, respectively [17].

### Frontier ASPECTS

The syngo.via Frontier ASPECTS prototype software (version 2.0.1, Siemens Healthcare GmbH, Erlangen, Germany) allows analyzing NCCT scans for early ischemic changes in acute stroke in the territory of the middle cerebral arteries. A probabilistic atlas has been created based on 150 normal NCCT datasets in which ASPECTS regions (caudate nucleus (CN), internal capsule (IC), insula (INS), lentiform nucleus (LN), and 6 regions in the vascular territory of the middle cerebral artery (M1–M6)) were segmented. This human brain atlas consists of ten volumes of interest for each brain hemisphere which represent the 10 ASPECTS regions. After automatically fitting of the atlas to an NCCT brain, likelihood for belonging to a specific ASPECTS region is appointed to every voxel. The likelihood of every voxel translates to the weight of the voxel-specific HU for computing the mean HU of every ASPECTS region. To exclude cerebrospinal fluid, old infarcts, bone and calcifications, and voxels that are either too dark (below 10 HU) or too bright (above 55 HU) are excluded.

The relative difference in mean HU between the individual ASPECTS region in the affected hemisphere and the contralateral hemisphere is computed and presented as a percentage HU difference. By using a predefined threshold for the relative HU difference, each ASPECTS region in the affected hemisphere is classified as affected (ischemic changes detected by the software) or not affected. The number of affected regions is used to calculate an ASPECT score. ASPECTS region–specific threshold values are used for the classification into ischemic and non-ischemic ASPECTS regions. The default threshold values were based on initial evaluations in patients in which the automated ASPECTS was optimized with CT perfusion–based infarct core assessment as reference standard [18]. The affected cerebral hemisphere is selected automatically by the software. If needed, the automated assessed affected hemisphere side can be adjusted to match the clinically affected cerebral hemisphere.

### Statistical analysis

To define the optimal threshold values for the relative HU change in the ASPECTS regions and to validate the findings, the included patients were divided in a training set (proportion of whole dataset 0.2) and a test set (proportion of whole dataset 0.8) using stratified random sampling. The datasets were stratified for affected ASPECTS regions (CN, IC, INS, LN, M1–M6) and for CT-scanner manufacturer. The training set and test set were assessed for significant differences in age, sex, NIHSS at baseline, stroke side, ischemic stroke history, prestroke modified Rankin Scale, and reference standard ASPECTS using *t* tests and chi-squared tests.

A specificity of ≥ 90% was used in the training set to find the software threshold settings with the optimal correlation between the computed ASPECTS and the reference standard to detect ischemic changes (Online Resource). Receiver-operating characteristic curves were created to calculate the area under the curve and to assess the sensitivity and specificity of the computed ASPECTS to detect ischemic changes in the test set with the thresholds defined in the training set. Bland-Altman plots were created to evaluate for systematic differences between software and reference standard.

The performance of computed ASPECTS and individual observers (1 and 2) in comparison to the reference standard as well as interobserver agreement was assessed with the intraclass correlation coefficient (ICC) using a one-way random-effects, absolute agreement, single-rater/measurement model (ICC[1,1]). The strictest ICC model was used because a selection of readers out of a panel of multiple expert readers assessed the ASPECTS [19]. For the ICC, values less than 0.5 are indicative of poor reliability, values between 0.5 and 0.75 indicate moderate reliability, values between 0.75 and 0.9 indicate good reliability, and values greater than 0.90 indicate excellent reliability. In addition, agreement per region (normal and abnormal) and agreement for trichotomized ASPECTS (0–4, 5–7, 8–10) were reported. All analyses were performed with the use of the SPSS software package, version 24.0.0.1 and R, version 3.5.1.

## Results

### Patients

**Fig. 1.**
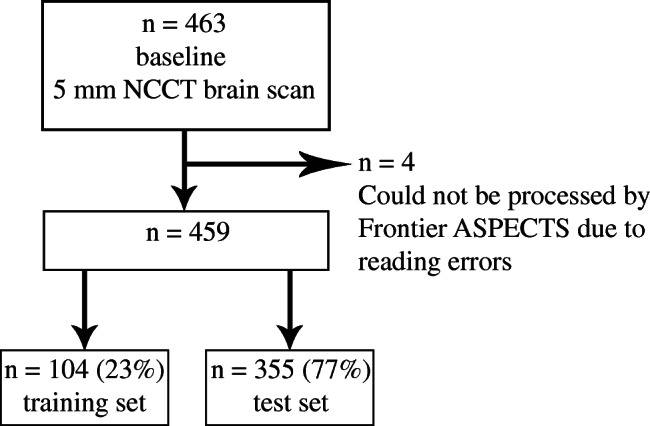
Flow chart of patients included in analysis

From the MR CLEAN trial, 463 patients had a baseline NCCT with a 5-mm slice thickness. The NCCTs of 4 patients could not be processed by the automated software due to reading errors, leaving 459 (> 99%) patients available for analysis (Fig. 1). For those 459 patients, 18 different scanners from 4 different manufacturers were used for NCCT acquisition (Online Resource, Table 1).

**Table 1:** Patient characteristics in the training set and test set

Characteristics	Training set (*n* = 104)	Test set (*n* = 355)
Age - Median (Interquartile range)	64.5 (54.3–77.0)	66 (54.0–76.0)
Male sex no. (%)	62 (59.6)	204 (57.5)
NIHSS^a^ score Median (interquartile range)	17 (13–21)	18 (15–22)
Location of stroke in left hemisphere - no. (%)	52 (50.0)	190 (53.5)
History of ischemic stroke - no. (%)	8 (7.7)	37 (10.4)
Prestroke modified Rankin scale score - no. (%)		
0	83 (79.8)	288 (81.1)
1	9 (8.7)	37 (10.4)
2	7 (6.7)	17 (4.5)
> 2	5 (4.8)	14 (3.9)
Duration (minutes) from stroke onset to imaging – Median (Interquartile range)	109 (65–201)	114 (68–196)
Reference standard ASPECTS		
Median (interquartile range)	8 (7–9)	7 (6–9)
0 (%)	1 (1)	1 (0)
1 (%)	0 (0)	3 (1)
2 (%)	0 (0)	3 (1)
3 (%)	1 (1)	10 (3)
4 (%)	3 (3)	18 (5)
5 (%)	6 (6)	37 (10)
6 (%)	13 (13)	36 (10)
7 (%)	25 (24)	72 (20)
8 (%)	25 (24)	80 (23)
9 (%)	16 (15)	52 (15)
10 (%)	14 (14)	43 (12)

^a^National Institutes of Health Stroke Scale

The affected cerebral hemisphere in Frontier ASPECTS matched the clinically affected hemisphere in 86% of the patients. The remaining scans (65) were adjusted to match the clinically affected brain hemisphere. Twenty-four of the 65 wrong classifications (37%) had an ASPECTS of 7–8, and 36 (55%) had an ASPECTS of 9–10 (Online Resource, Table 2). However, after correcting for the affected hemisphere on imaging, the discriminating performance did not improve significantly (Online Resource, Figure 2; Online Resource, Table 4). Stratified random sampling resulted in allocation of 104 patients (23%) to the training set and allocation of 355 patients (77%) to the test set. Due to stratification limitations, this division was not exactly a 20%/80% division. No significant differences were found between the training set and test set (Table 1).

**Table 2:** Performance of Frontier ASPECTS in the test set in comparison to reference standard with optimal thresholds defined in the training set

ASPECTS region	Threshold value (%)	Specificity (%)	Sensitivity (%)	TP	TN	FP	FN	AUC	(95% CI)
CN, IC, INS, LN	5.6	89	57	353	711	87	269	0.795	(0.771–0.819)
M1–M6	4.7	89	41	154	1565	191	220	0.750	(0.722–0.778)
Combined		89	51	507	2276	278	489		
All 10 regions	4.9	89	54	533	2274	280	463	0.713	(0.692–0.733)

### Training set

In the training set (*n* = 104), with software thresholds for a specificity of ≥ 90%, we found a sensitivity of 33–49% and an area under the curve (AUC) of 0.741–0.785 for detection of an affected ASPECTS region (Online Resource, table 4).

### Validation with test set

The area under the curve in the test set for assessment of ischemic changes in the central regions (caudate, insular ribbon, internal capsule, and lentiform nucleus) and the cortical regions (M1–M6) was similar to the area under the curve in the training set (Table 2). With the optimal threshold value from the training set for detection of ischemic changes in the central regions (5.6%), a specificity and sensitivity of 89% and 57% were found in the test set. With the optimal threshold value from the training set for detection of ischemic changes in the cortical regions (4.7%), a specificity and sensitivity of 89% and 41% were found in the test set (Table 2). Overall computed ASPECTS and reference standard agreed on region normality in 2276 regions and on the presence of ischemia in 507 regions which resulted in an overall accuracy of 78%. The accuracy of trichotomized computed ASPECTS was 60%. The ICC for the full range of ASPECTS was 0.526 (0.447–0.597). Similar results were obtained with 1 threshold for all ASPECTS regions. The Bland-Altman plot for ASPECTS difference between automated ASPECTS and reference standard showed a mean difference of 0.59 (95% CI − 3.20–4.39) (Online Resource, table 5 and figure 2). The default region–specific thresholds resulted in a lower ICC than the optimized thresholds.

No differences were found in area under the curve in evaluating ASPECTS regions in scans from different vendors (Online Resource, Table 3).

**Table 3:** Agreement comparison of Frontier ASPECTS and observers and agreement between observers in the test set (*n* = 355)

	Agreement on normal regions (*n*)	Agreement on abnormal regions (*n*)	Overall agreement (%)	Trichotomized^a^ ASPECTS agreement (%)	ASPECTS ICC (95% CI)
Frontier ASPECTS (default thresholds) vs. reference standard	2046	613	2785 (78)	200/355 (56)	.494 (.412–.569)
Frontier ASPECTS (optimized thresholds for [CN/ IC/ INS/ LN] & [M1-M6 regions]) vs. reference standard	2276	507	2783 (78)	213/355 (60)	.526 (.447–.597)
Frontier ASPECTS (1 optimized threshold for all 10 regions) vs. reference standard	2274	533	2807 (79)	210/355 (59)	.537 (.459–.607)
observer 1 vs. reference standard	2430	536	2742 (84)	212/355 (60)	.464 (.378–.542)
observer 2 vs. reference standard	2353	389	2742 (77)	204/355 (57)	.383 (.291–.468)
observer 1 vs. observer 2	2713	337	3050 (86)	238/273 (77)	.667 (.605–.721)

^a^classification by ASPECTS 0–4, 5–7 and 8–10

### Comparison of computed ASPECTS to observers ASPECTS

The agreement per region of computed ASPECTS with the reference standard was similar to the region agreement of the observers with the reference standard (78% and 79% versus 84% and 77%). The agreement for trichotomized ASPECTS for the computed ASPECTS and the observers was 60% and 59% versus 60% and 57% (Table 3).

Comparison of the agreement of computed ASPECTS with the reference standard showed an ICC of 0.526 (95% CI 0.447–0.597) with two optimized thresholds for [CN/IC/INS/LN] and [M1–M6 regions] and an ICC 0.537 (95% CI 0.459–0.607) with one optimized threshold for all 10 regions. Comparison of the agreement of observers with the reference standard showed lower ICCs of 0.464 (95% CI 0.378–0.542) and 0.383 (95% CI 0.291–0.468).

The agreement between observers (overall agreement 86% and ICC 0.667 (95% CI 0.605–0.721)) was higher than the agreement between computed ASPECTS and the reference standard (Table 3).

## Discussion

In this study, we evaluated the performance of Frontier ASPECTS software for the detection of early ischemic brain changes on NCCT scans. Our study showed a moderate agreement between Frontier ASPECTS and the reference standard, defined as the consensus between two expert readers. The agreement was similar to the agreement between individual readers and the reference standard, but less than interobserver agreement. Frontiers ASPECTS could aid in visual evaluation of the NCCT for the detection and quantification of early ischemic changes due to acute ischemic stroke.

The Frontier ASPECTS software has specific advantages. The exclusion of bone and old infarcts before assessing the mean density of the ASPECTS regions improves the accuracy of the brain parenchyma density measurements. Old brain infarcts were found in approximately 20% of the NCCT scans and should not be used in the assessment of ASPECTS. The calculation of the mean density of the regions and the relative change in density enables adaption of the threshold to optimize the tool for specific contexts and allows the future use of the relative density values instead of the dichotomous outcomes (normal/abnormal) in prediction models. Machine learning model algorithms trained on dichotomous outcome will lack this opportunity [3, 20, 21]. Current disadvantages are the reading errors in a minority of scans and the mismatch in the assessment of the affected hemisphere. However, the latter can be corrected manually, which will result in a correct assessment of the ASPECTS. Although recent evidence showed a considerably lower performance of Frontier ASPECTS, this difference could be explained by our threshold optimization and the use of software version 2.0.1 instead of 1.2.0. [12].

A major problem in evaluation of automated software to support clinical validation is the choice of reference standard. Previous studies have used as reference standard the DWI performed within a specified time frame of the NCCT [3, 20, 21], follow up NCCT [22, 23], or MRI [24]. In studies in which follow-up NCCT was used as reference, only lesions that were present on base line and follow-up imaging were scored [22], or the definite final infarct core on follow-up NCCT was scored [23]. DWI-ASPECTS as reference standard could be problematic due to the potential time delay between test and reference standard of up to 2 h which could affect the results. More important are the differences in the underlying signal changes. The NCCT scan in the early phase could be completely normal when cytotoxic edema has resulted in an abnormal DWI signal. The NCCT scan will only reveal density decrease in the next phase of vasogenic edema. This might result in low sensitivity of the algorithm in the early phase after the event. Follow-up imaging as reference standard ignores the effects of intravenous thrombolysis and endovascular thrombectomy. Minor changes on NCCT could be reversible after treatment which could result in a low specificity of the algorithm [23].

Comparing ASPECTS with perfusion changes could produce valuable information, but it would require a different approach, as abnormalities in perfusion maps, which are apparent immediately after stroke onset, do not necessarily lead to imaging abnormalities on NCCT. However, given the relevance of ASPECTS scoring for ischemic stroke treatment and the issues with human ASPECTS scoring like moderate observer agreement, the purpose of this manuscript is to assess whether software can be used to reliably automate the ASPECTS scoring and support clinicians in assessing early ischemic changes. Therefore, comparing with DWI or perfusion imaging is beyond the aim of this paper.

We therefore used consensus readings of expert observers as reference standard. The only disadvantage could be that observers are not able to detect subtle abnormalities on NCCT scans which potentially could be detected by density measurements or machine learning techniques. Therefore, we aimed at a comparison with readers as the current context of automated image analysis is the support and potential replacement of a reader in order to increase the robustness of evaluation.

Independent of the approach, we found other studies reporting a similar performance. When we compare the performance of Frontiers ASPECTS with Brainomix e-ASPECTS, similar sensitivity (54% vs 44–46%) and specificity (89% vs 91–94%) was found [3, 22]. In addition, the performance of automated ASPECTS was better than observers [3, 22]. A similar study analyzing relative Hounsfield unit density per region found a sensitivity of 45% and a specificity of 93% for a HU ratio threshold of < 0.94 and an area under the curve of 0.780 [24]. A study assessing RAPID@IschemaView automated ASPECTS found a performance equal to the agreement read of expert neuroradiologists [25].

Finally, a machine learning algorithm with DWI-ASPECTS as reference standard resulted in ICC of 0.76, a sensitivity of 66%, and a specificity of 92%. This study, similar to the current study, optimized the algorithm on a training set and tested the performance on a test set [20].

The strength of this study includes the heterogeneity of the included patients and the use of 18 different scanner types from four major CT-scanner vendors which enables a reliable translation of the study results into clinical practice. Second, the rigorous methodology with subdivision in training and test set and the multiple metrics to assess the accuracy is a strength. Third, most studies assessing ASPECTS software performance with baseline ASPECTS as reference standard use the ASPECTS assessed by observers for both consensus for the reference standard as for individual observer analysis, which created a bias in the analysis of observer agreement [9, 10, 12]. To prevent this bias in our analyses, we strictly split the expert readers panel, using independent expert readers for the reference standard consensus and for individual observer analysis.

A possible limitation of this study is the use of vendor-specific software, since the software could work better for CT-scans acquired on Siemens equipment. However, the discriminating performance of the software in ASPECTS regions did not differ significantly between CT-scans acquired on Siemens CT-scanners and CT-scanners of other vendors. Secondly, in our study, we used a panel of observers to provide ASPECTS, representing the variability in observers for ASPECTS in clinical practice. Most studies do not describe the use of a panel of observers to provide ASPECTS, nor does any author describe the type of ICC they use. A practical limitation is the use of a fixed specificity of ≥ 90% in the analyses. The shape of the receiver-operating characteristic curves in this study suggests a better discriminative ability between affected and unaffected ASPECTS regions for a lower specificity, and one could want to use another threshold values resulting in a change in specificity or sensitivity, depending on their context and the type of data.

This software tool should not be intended as replacement of the physician. We would recommend seeing this software tool in clinical practice as an aid for physicians. Further research is needed to compare the performance of this ASPECTS software to software of other vendors. Besides this, the role of this ASPECTS software in clinical practice needs to be established by evaluating the added value in predicting outcome compared with ASPECTS based on readers.

## Conclusion

In conclusion, the performance of Frontier ASPECTS is comparable to expert readers and is able to support readers in the detection of early ischemic changes in a standardized way.

## Electronic supplementary material

ESM 1 (DOCX 202 kb)
