# Supplementary material for: Validation of automated Alberta Stroke Program Early CT Score (ASPECTS) software for detection of early ischemic changes on non-contrast brain CT scans
Source: Neuroradiology. 2020 Aug 28;63(4):491–8. doi: 10.1007/s00234-020-02533-6 (PMC7966210; doi:10.1007/s00234-020-02533-6)
Supplement: Supplementary file 1 — (DOCX 202 kb) [file 234_2020_2533_MOESM1_ESM.docx]

**SUPPLEMENTAL MATERIAL**

**Data supplement**

| **Suppl. table 1.** CT-scanner types used for imaging in the included patients | | |
| --- | --- | --- |
| **Vendor** | **CT-scanner type** | **Patients (n)** |
| Philips | iCT 256 | 89 |
| Philips | Brilliance 16 | 1 |
| Philips | Brilliance 40 | 44 |
| Philips | Brilliance 64 | 31 |
| Philips | Gemini TF TOF 64 | 1 |
| Philips | Mx8000 IDT 16 | 1 |
| Siemens | Somatom Definition Flash | 62 |
| Siemens | Somatom Definition AS+ | 24 |
| Siemens | Somatom Definition AS | 7 |
| Siemens | Biograph 64 | 3 |
| Siemens | Somatom Sensation 16 | 1 |
| Siemens | Somatom Sensation 64 | 66 |
| GE | LightSpeed VCT | 35 |
| GE | BrightSpeed S | 9 |
| GE | Discovery CT750 HD | 2 |
| Toshiba | Aquilion | 18 |
| Toshiba | Aquilion ONE | 64 |
| Toshiba | Aquilion PRIME | 1 |
| **Total** | 18 | **459** |

| **Suppl. table 2.** Affected hemisphere detection accuracy of computed ASPECTS | | |
| --- | --- | --- |
| Reference standard ASPECTS | Correct hemisphere (%) |  |
| 0 | 2/2 (100) |  |
| 1 | 3/3 (100) |  |
| 2 | 3/3 (100) |  |
| 3 | 11/11 (100) |  |
| 4 | 20/21 (95) |  |
| 5 | 43/43 (100) |  |
| 6 | 45/49 (92) |  |
| 7 | 87/97 (90) |  |
| 8 | 91/105 (87) |  |
| 9 | 56/68 (82) |  |
| 10 | 33/57 (58) |  |
| Total | 394/459 (86) |  |
|  | | |

| **Suppl. table 3.** Influence of correcting the affected hemisphere on the software performance in NCCT baseline imaging ASPECTS regions. | | | |
| --- | --- | --- | --- |
| Test result variables | AUC | 95% CI | Std. Error^*^ |
| Corrected hemisphere | 0.789 | 0.771-0.807 | 0.009 |
| Software hemisphere | 0.764 | 0.745-0.783 | 0.010 |

**Training set methods**

Three approaches were evaluated to define the optimal thresholds for detection of early ischemic changes in the training set. Firstly, the thresholds for every separate ASPECTS region were calculated; secondly, one threshold for the central regions (caudate, insular ribbon, internal capsule and lentiform nucleus) and one threshold for the cortical regions (M1-M6), and thirdly one threshold for all 10 ASPECTS regions. For each analysis receiver-operating characteristic (ROC) curves were created to calculate the area under the curve, and to assess the sensitivity for the optimal threshold, which was defined as a threshold which results in a specificity of ≥ 90% for the computed ASPECTS to detect ischemic changes.
Based on the results of the training set, only the second and third approach were used and the thresholds were validated in the test set to provide an unbiased evaluation of the performance of optimal thresholds in computed ASPECTS by comparison to the reference standard.

**Training set results**

The optimal threshold values per region to assess ischemia with a specificity of ≥ 90% ranged from 2.8% for the M3 region to 7.4% for the caudate nucleus (Table 2). The area under the curve ranged from .510 to .851. Comparison of the computed ASPECTS to the reference standard, while using these threshold values, resulted in an ICC of .394 (95% CI .220-.545).
The optimal threshold for the combined central regions (caudate, insular ribbon, internal capsule and lentiform nucleus) was 5.6%, with a corresponding specificity and sensitivity of 90% and 49%, respectively. The area under the curve was .785 (Table 2). The optimal threshold for the combined remaining M1-M6 regions was 4.7%, with a corresponding specificity and sensitivity of 90% and 33%, respectively. The area under the curve was .741 (Table 2). Comparison of the computed ASPECTS to the reference standard while using these threshold values resulted in an ICC of .356 (95% CI .177-.513).
When applying the same thresholds for all ASPECTS regions, the optimal threshold was 4.9% and the area under the curve was .794 (95% CI .761-.826) (Table 2). Comparison of the computed ASPECTS to the reference standard, while using this threshold, resulted in an ICC of .372 (95% CI .177-.513).
Affected M3 and M6 regions were hardly present in the training set and the approach with region-specific thresholds was not validated in the test set.

| **Suppl. table 4.** ASPECTS region threshold values for Frontier ASPECTS and performance in the training set (n = 104). | | | | | | | | | |
| --- | --- | --- | --- | --- | --- | --- | --- | --- | --- |
| **ASPECTS region** | **Threshold value, %** | **Specificity, %** | **Sensitivity, %** | **TP** | **TN** | **FP** | **FM** | **Area under the curve** | **(95% CI)** |
| CN | 7.4 | 90 | 43 | 18 | 56 | 6 | 24 | .741 | (.643-.840) |
| INS | 6.2 | 91 | 29 | 21 | 29 | 3 | 51 | .709 | (.601-.818) |
| IC | 4.2 | 90 | 67 | 2 | 91 | 10 | 1 | .736 | (.426-1.00) |
| LN | 5.4 | 91 | 67 | 48 | 29 | 3 | 24 | .851 | (.779-.922) |
| M1 | 5.8 | 91 | 32 | 6 | 77 | 8 | 13 | .701 | (.582-.821) |
| M2 | 4.9 | 90 | 27 | 6 | 74 | 8 | 16 | .653 | (.513-.792) |
| M3 | 2.8 | 90 | 0 | 0 | 92 | 10 | 2 | .686 | (.480-.893) |
| M4 | 5.1 | 91 | 21 | 3 | 82 | 8 | 11 | .797 | (.682-.912) |
| M5 | 4.8 | 90 | 60 | 6 | 85 | 9 | 4 | .803 | (.660-.946) |
| M6 | 4.1 | 90 | 0 | 0 | 92 | 10 | 2 | .510 | (.410-.610) |
| CN, IC, INS, LN | 5.6 | 90 | 49 | 92 | 205 | 22 | 97 | .785 | (.740-.829) |
| M1-M6 | 4.7 | 90 | 33 | 23 | 500 | 55 | 46 | .741 | (.679-.802) |
| All regions | 4.9 | 90 | 49 | 126 | 705 | 77 | 132 | .794 | (.761-.826) |
| ASPECTS indicates Alberta Stroke Program Early CT Score; CN, caudate nucleus; INS, insular ribbon; IC, internal capsule; LN, lentiform nucleus; MCA, middle cerebral artery; M1, anterior MCA cortex; M2, MCA cortex lateral to insular ribbon; M3, posterior MCA cortex; M4, M5, and M6 are anterior, lateral, and posterior MCA territories immediately superior to M1, M2, and M3, rostral to basal ganglia; TP, true positive; TN, true negative; FP, false positive; FN, false negative. | | | | | | | | | |

| **Suppl. table 5.** Error matrix test set | | | | | | | | | | | | | |
| --- | --- | --- | --- | --- | --- | --- | --- | --- | --- | --- | --- | --- | --- |
|  |  |  |  |  |  | **Reference standard ASPECTS** | | |  |  |  |  |  |
|  |  | 0 | 1 | 2 | 3 | 4 | 5 | 6 | 7 | 8 | 9 | 10 | **total** |
| **Software ASPECTS*** | 0 | 0 | 0 | 0 | 0 | 0 | 0 | 0 | 0 | 0 | 0 | 0 | 0 |
|  | 1 | 0 | 0 | 0 | 2 | 0 | 0 | 0 | 0 | 0 | 0 | 0 | 2 |
|  | 2 | 1 | 0 | 0 | 0 | 2 | 0 | 0 | 0 | 1 | 1 | 0 | 5 |
|  | 3 | 0 | 1 | 2 | 1 | 1 | 1 | 0 | 4 | 0 | 1 | 0 | 11 |
|  | 4 | 0 | 1 | 0 | 2 | 3 | 4 | 4 | 4 | 0 | 0 | 1 | 19 |
|  | 5 | 0 | 1 | 0 | 1 | 2 | 5 | 1 | 3 | 0 | 1 | 0 | 14 |
|  | 6 | 0 | 0 | 0 | 2 | 5 | 10 | 4 | 6 | 4 | 5 | 0 | 36 |
|  | 7 | 0 | 0 | 0 | 1 | 1 | 7 | 6 | 10 | 10 | 4 | 2 | 41 |
|  | 8 | 0 | 0 | 0 | 1 | 3 | 4 | 7 | 18 | 23 | 9 | 4 | 69 |
|  | 9 | 0 | 0 | 0 | 0 | 0 | 4 | 9 | 14 | 19 | 9 | 6 | 61 |
|  | 10 | 0 | 0 | 1 | 0 | 1 | 2 | 5 | 13 | 23 | 22 | 30 | 97 |
|  | **total** | 1 | 3 | 3 | 10 | 18 | 37 | 36 | 72 | 80 | 52 | 43 | 355 |
|  | *Threshold of 5.6% for combined central regions (caudate, insular ribbon, internal capsule and lentiform nucleus) and 4.7% for cortical regions (M1-M6). | | | | | | | | | | | | |

| **Suppl. table 6.** Performance in ASPECTS regions of CT-scanner vendors. | | | | |
| --- | --- | --- | --- | --- |
| CT-scanner vendor | patients (n) | AUC | 95% CI |  |
| Philips | 126 | .791 | .763-.819 |  |
| Siemens | 127 | .781 | .749-.813 |  |
| GE | 37 | .809 | .758-.860 |  |
| Toshiba | 65 | .791 | .750-.833 |  |
| AUC, Area Under the Curve. | | | |  |

| **Suppl. figure 1.** Influence of correcting the affected hemisphere on the software performance in NCCT baseline imaging ASPECTS regions. |
| --- |
| 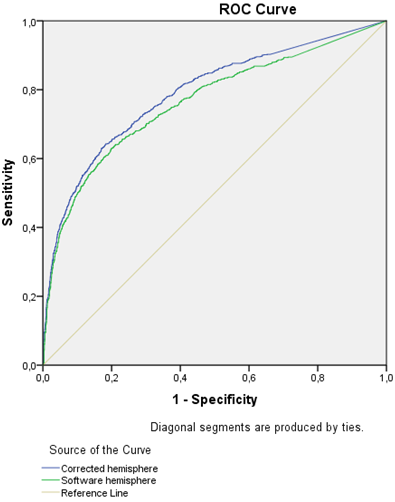 |

| **Suppl. figure 2.** Bland-Altman plots for ASPECTS difference between automated ASPECTS and reference standard. |
| --- |
| 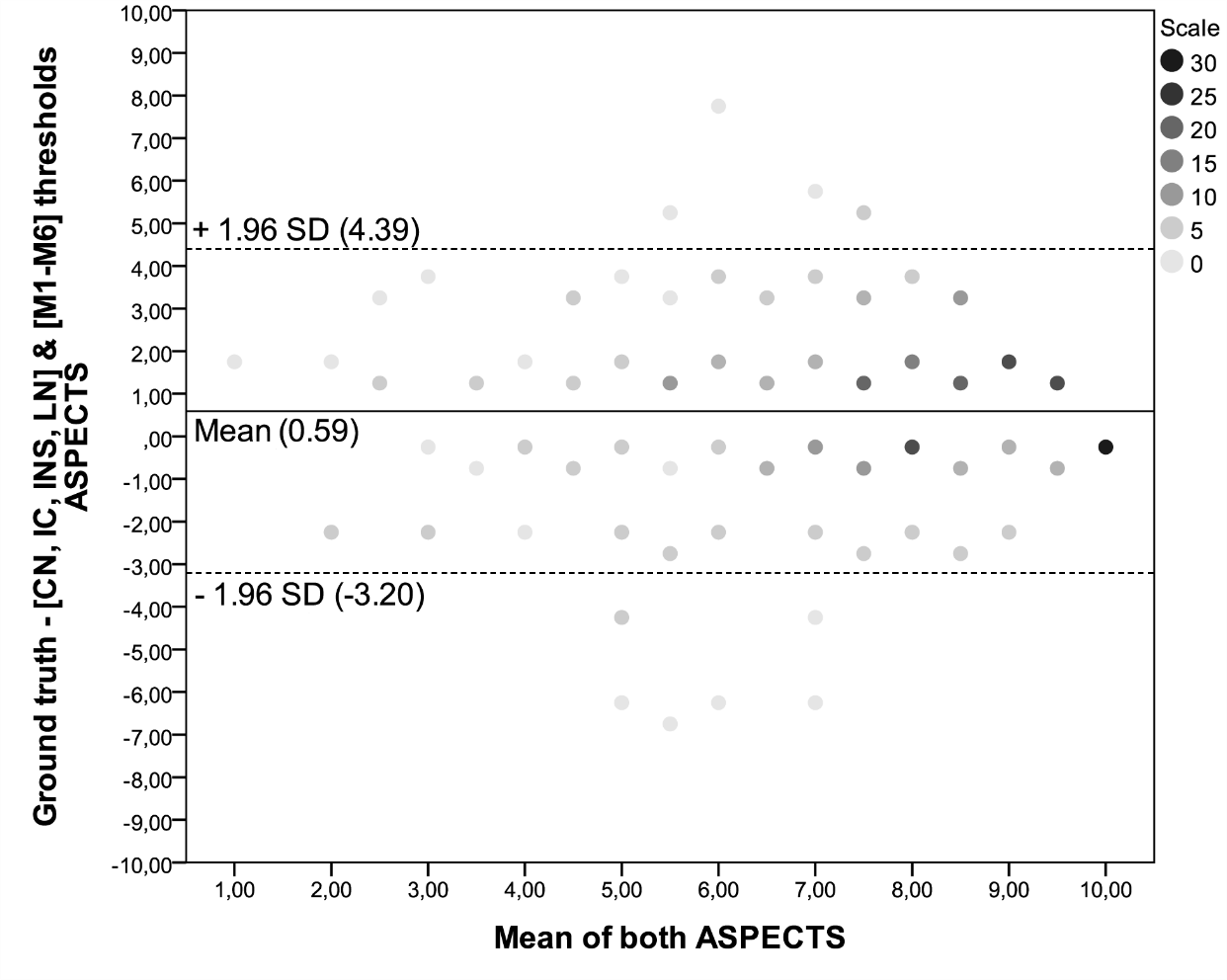 |
